# A modification of retrograde Langendorff‐perfusion to increase the yield of Ca^2+^-tolerant murine atrial cardiomyocytes isolated from remodelled, fibrotic atria

**DOI:** 10.3389/fphar.2026.1836453

**Published:** 2026-07-08

**Authors:** Jan Peter Reinhardt, Yasmina Steins, Luca Bartolomeo Tardio, Max Gieske, Paul Pauls, Matthias Dodo Seidl, Uwe Kirchhefer, Lars Eckardt, Frank Ulrich Müller, Jan Sebastian Schulte

**Affiliations:** 1 Institute of Pharmacology and Toxicology, University of Münster, Münster, Germany; 2 Department of Cardiology II - Electrophysiology, University Hospital Münster, Münster, Germany

**Keywords:** atrial cardiomyocyte isolation, cardiac remodelling, cell isolation, cellular electrophysiology, langendorff retrograde perfusion, mouse heart

## Abstract

**Objective:**

Langendorff-perfusion is an established method to isolate cardiomyocytes from cardiac tissue for the investigation of cellular remodelling in cardiovascular diseases. Despite the existence of several protocols for isolation of murine atrial cardiomyocytes (aCM), extraction of a sufficient yield of Ca^2+^-tolerant aCM from remodelled, fibrotic murine atria with established protocols remains challenging. Here, we evaluate if a novel, simple modification of an established Langendorff-based protocol improves the isolation of aCM.

**Methods:**

The effect of an additional ventricular ligature during Langendorff-perfusion on the yield of isolated aCM was evaluated in mice with cardiomyocyte-specific overexpression of CREM-IbΔC-X, a mouse model of pronounced atrial dilatation and spontaneous onset atrial fibrillation compared to littermate controls.

**Results:**

Atria with overexpression of CREM-IbΔC-X exhibited dilatation, increased mass and enhanced fibrosis. From such remodelled atria, we observed significantly lower extractions both of total and morphologically intact aCM compared to wildtype. The additional ventricular ligature did not affect total aCM yield in wildtype, but in fibrotic atria; the total aCM count was significantly increased (26 ± 4 vs. 15 ± 3 aCM/10 µL). Importantly, the number of morphologically intact, Ca^2+^-tolerant aCM available for functional assessment from fibrotic atria was doubled with the modified protocol.

**Conclusion:**

The modification of an established Langendorff-perfusion protocol for aCM isolation by an additional ventricular ligature is associated with a numerically higher yield of morphologically intact, Ca^2+^-tolerant aCM from remodelled atria. This may facilitate research on cellular electrophysiology in cardiomyocytes of murine models with atrial remodelling.

## 1 Introduction

Cardiovascular diseases are a significant contributor to the global disease burden (Roth et al., 2020). Despite extensive research efforts, the mechanisms of progressive structural changes in many cardiovascular diseases, commonly referred to as “remodelling”, remain not fully understood. For atrial fibrillation cellular and tissue remodelling that lead to atrial cardiomyopathy are driving factors for its progressive pathophysiology (Schotten et al., 2011; Nattel and Dobrev, 2016; Nattel et al., 2020). Consequently, this remodelling is of scientific interest as a potential therapeutic target for the management of atrial fibrillation (Linz et al., 2024) and many research groups are investigating its underlying mechanisms in a variety of experimental models.

The isolation of individual, primary cardiomyocytes (CM) is a critical method for the investigation of cellular remodelling in cardiovascular diseases. A variety of protocols for the isolation of atrial (aCM) and ventricular CM (vCM) from different model organisms or human samples have been published and are widely utilised. Many protocols for the isolation of CM from animal models involve the retrograde perfusion of the heart with digestive enzymes based on a technique first described by Oscar Langendorff in 1895 (Langendorff, 1895; Berry et al., 1970; Gould and Powell, 1972) which is also used for studying electrophysiologic mechanisms of arrhythmias (Johna et al., 1998; Milberg et al., 2007). Retrograde perfusion requires thorough preparation of the heart and cannulation of the aorta, which is a technically demanding procedure, particularly in small model organisms such as mice.

Importantly, most perfusion protocols use perfusion solutions that are free of calcium (Ca^2+^) or have reduced Ca^2+^-concentrations compared to physiological extracellular Ca^2+^-concentrations (Lemoine et al., 2011; Voigt et al., 2015; Tomsits et al., 2020; Isenberg and Klockner, 1982). The isolated CM are subsequently re-exposed to physiological Ca^2+^-concentrations to enable functional assessment. However, a part of the CM becomes irreversibly hypercontractile and dies after re-exposure to Ca^2+^ depending on the CM yield and quality of the isolation (Vahouny et al., 1970; Berry et al., 1970). This phenomenon is commonly referred to as the “Ca^2+^-paradox” (Powell, 1983; Zimmerman and Hülsmann, 1966).

Low CM yields increase the number of experiments and pose a challenge to the reproducibility of experiments. Thus, the aim is to isolate as many rod-shaped, striated, and Ca^2+^-tolerant myocytes as possible for subsequent functional analyses (Cheung et al., 1984). For this purpose a variety of modified protocols have been developed, and modern, visual training material is available (Tomsits et al., 2020; Bode et al., 2018; Sharpe et al., 2016). Nevertheless, isolating Ca^2+^-tolerant aCM, especially from remodelled, fibrotic atria, remains challenging. One well established animal model with severe atrial remodelling and atrial fibrillation is the CREM-IbΔC-X mouse model (Müller et al., 2005; Keefe et al., 2023; Riley et al., 2012; Bukowska et al., 2018; Stümpel et al., 2018; Seidl et al., 2017; Yao et al., 2018; Schulte et al., 2016; Li et al., 2014; Kirchhof et al., 2013; Chelu et al., 2009).

In this study, we developed and evaluated a modification of the Langendorff-method, which resulted in enhanced aCM yield from fibrotic, remodelled atria of the CREM-IbΔC-X-mouse model of atrial fibrillation.

## 2 Methods

### 2.1 Animals

FVB/N mice with heterozygous, αMHC-promotor-driven expression of Cre recombinase (Liffers et al., 2025; Agah et al., 1997) (CTR) were crossbred with mice overexpressing the CREM-isoform IbΔC-X under control of the αMHC-promotor (Müller et al., 2005) (TG). CTR and TG littermates were analysed at an age of 12 weeks. The applied experimental methods confirmed to the instructions of Directive 2010/63/EU of the European Parliament on the protection of animals used for scientific purposes and were approved by the local authorities (Landesamt für Verbraucherschutz und Ernährung NRW; permissions: 53.5.32.7.1/MS-07842, 84–02.05.50.15.024, 81–02.05.50.20.012).

### 2.2 Study design

**FIGURE 1:**
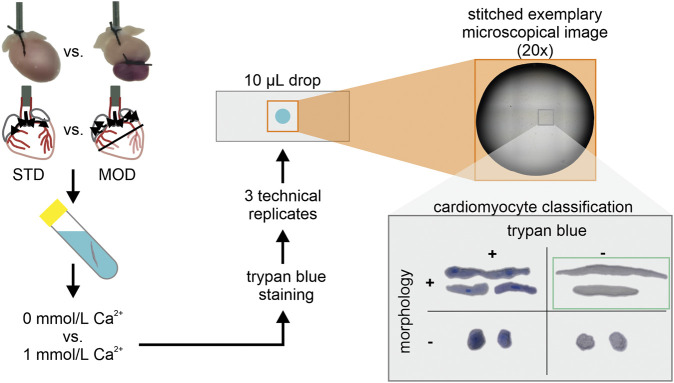
Overview of study design. Isolations of aCM were performed according to standard protocol (STD) and with additional ventricular ligature (MOD) (exemplary images and schematic views of heart perfusion shown) for each 3 animals of CTR and TG group. aCM suspension was examined both at 0 and 1 mmol/L Ca^2+^ after trypan blue staining of each 3 technical replicates. 10 μL of stained aCM suspension were placed on a slide and a microscopic image was acquired of the whole aCM suspension drop (stitched exemplary microscopic image shown). aCM were counted and classified depending on morphological intactness and trypan blue uptake (exemplary aCM shown in table). Only aCM with intact morphology and without trypan blue uptake were considered eligible for functional single-cell assessment (green box). aCM: atrial cardiomyocytes, Ca^2+^: calcium, CTR: control group, TG: transgenic group (CREM-IbΔC-X mice), STD: standard isolation protocol, MOD: modified isolation protocol.

Animal handling and experimental procedures were performed in an unblinded fashion. All preparations, cannulations, and isolations were performed by the same experienced experimentalist. For image analysis, investigators were blinded to mouse genotype and experimental protocol used for myocyte isolation until the generation of graphs and statistics. All experiments were included in the analysis. Figure 1 represents an overview of the general study design as further explained in the following.

### 2.3 Morphological characterisation

Images of Langendorff-perfused hearts were acquired in front of a standardised scale and the 2D atrial area was measured using ImageJ software (Schindelin et al., 2012; Rueden et al., 2017). First, the body weight of sacrificed mice was measured. Thereafter, the hearts were removed, separated into atria and ventricles and weighed using an analytical balance.

**FIGURE 2:**
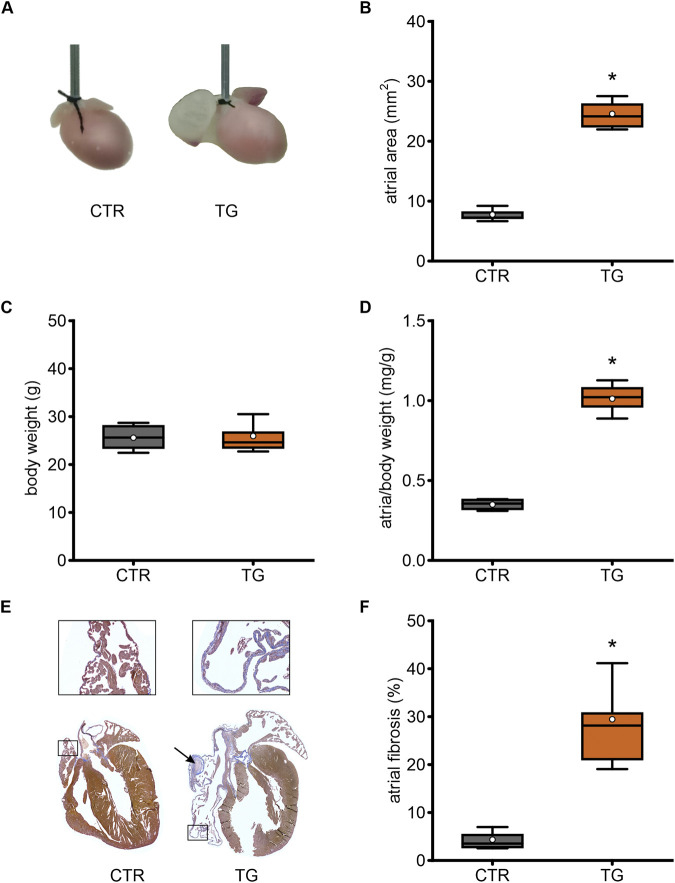
Extensive atrial remodelling in mice with CREM-IbΔC-X overexpression **(A)** Exemplary cannulated hearts. **(B)** 2D-area of perfused hearts: severe dilatation in TG hearts. **(C)** Body weight. **(D)** Relative atrial mass to body weight: raised in TG hearts. **(E)** Exemplary Masson-Trichrome-stained hearts with 5× magnified atrial wall sections, the arrow marks an atrial thrombus as often observed in TG atria. **(F)** Atrial fibrosis determined by proportion of blue stained tissue: enhanced atrial fibrosis in TG atria. (12 weeks, n = 6 per group, except for 2D-area: n = 5 per group, t-test *p < 0.05). CTR: control group, TG: transgenic group (CREM-IbΔC-X mice).

Atrial fibrosis was quantified in Masson-Trichrome-stained histological preparations as described before (Scholz et al., 2019) (exemplary images Figure 2E). Masson-Trichrome-stained paraffin sections were prepared using standard histological procedures. Whole-slide images were acquired with a microscope (Eclipse Ti-E, 10× objective). The proportion of blue-stained tissue was quantified using ImageJ software by relating the blue-positive area to the total tissue area within manually defined regions of interest only including the atrial musculature. Morphological characterisation was performed in a sex-balanced sample.

### 2.4 aCM isolation

**FIGURE 3:**
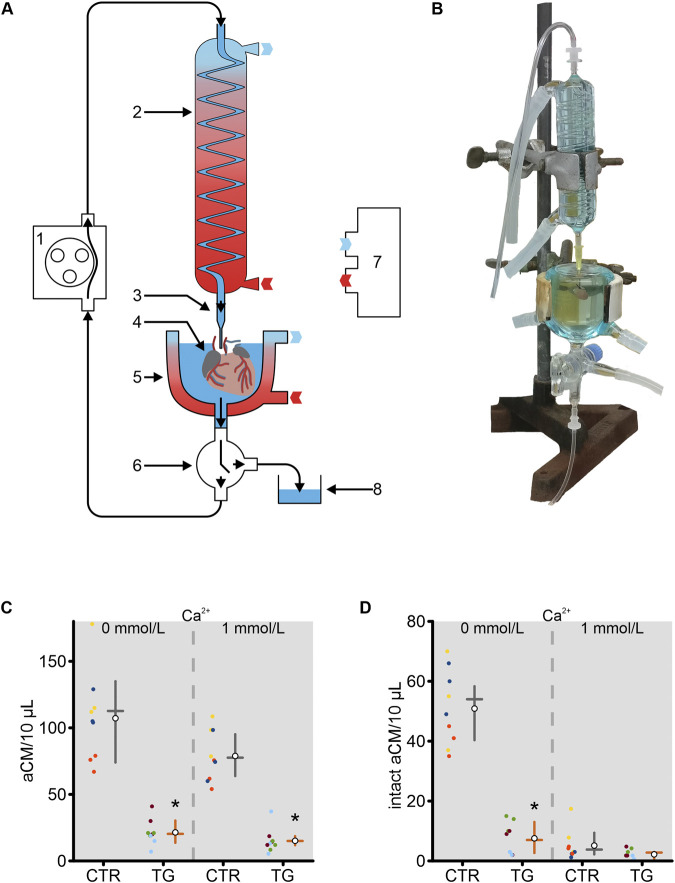
Reduced aCM yield with established Langendorff-perfusion protocol from remodelled atria **(A)** Schematic view of a Langendorff-perfusion-setup with components: 1) roll pump, 2) standard counterflow heat exchanger, 3) cannula, 4) perfused heart in 5) heated bath, 6) three-way stopcock for solution exchange, 7) heating circulator, 8) waste. **(B)** Exemplary picture of the used setup. **(C)** Total aCM yield per 10 µL: lower total aCM-extraction from TG vs. CTR atria. **(D)** Count of intact aCM per 10 µL: lower at 0 mmol/L Ca^2+^ in TG, also a tendency to lower yield of intact aCM from TG atria at 1 mmol/L Ca^2+^. (n = 3 vs. 3 per genotype, 3 technical replicates per isolation (raw data points, colour-coded per isolation), statistics on mean per isolation, t-test *p < 0.05 vs. CTR) aCM: atrial cardiomyocytes, CTR: control group, TG: transgenic group (CREM-IbΔC-X mice).

Atrial cardiomyocytes (aCM) were isolated via retrograde perfusion of the cannulated aorta (Langendorff-perfusion, (Langendorff, 1895)) from explanted hearts of 12 week old male mice. Throughout the preparation and cannulation process the hearts were submerged in cold Tyrode’s solution (Tyrode). After cannulation, the hearts were carefully flushed with 2 mL of heparinised (50 IU/mL) Tyrode and transferred to a modified Langendorff apparatus with a submerged heart as previously described (Motayagheni, 2017) (Figures 3A,B). The perfusion protocol was adapted from Lemoine et al. (Lemoine et al., 2011). Hearts were sequentially perfused with washing solution and Ca^2+^-free Tyrode (Table 1) for 3.5 min. Then, the hearts were perfused with collagenase-containing (Collagenase type II, Worthington Biochemical Corporation, Lakewood, NJ USA) digestive solution. The perfusion time with digestive solution was 30 min for CTR hearts and 32 min for TG hearts. To stop collagenase activity, perfusion with BSA-containing stop solution followed for another 3.5 min. Perfusion duration for the first and the last protocol steps were corrected for the apparatus-specific dead volume clearing time. After the perfusion protocol, the atria were separated from the ventricles and cut into pieces. Tissue pieces were carefully triturated in 5 mL cooled storage solution on ice (Table 1, modified from (Isenberg and Klockner, 1982)) for 5 min to dissolve isolated aCM from tissue. To test for Ca^2+^-tolerance, the generated aCM suspension was divided into two samples. Ca^2+^ was carefully added to one sample in a stepwise manner until a concentration of 1 mmol/L was reached (Table 2).

**TABLE 1:** Solutions.

Substance	Concentration (mmol/L)
Ca^2+^-free Tyrode’s solution (Tyrode, (Tyrode, 1910), modified)	
NaCl	136.0
KCl	5.4
MgCl_2_ x 6H_2_O	1.0
HEPES	5.0
NaH_2_PO_4_ x H_2_O	0.33
Glucose (added on day of experiment)	10.0
​	adjusted to pH 7.4 with KOH
Ca^2+^ Tyrode: Ca^2+^-free Tyrode plus	
CaCl_2_	1.0
Washing solution: Ca^2+^-free Tyrode plus	
CaCl_2_	2.0
Heparin (Heparin-sodium, ratiopharm, Germany)	50 IU/mL
Digestive solution: Ca^2+^-free Tyrode plus	
CaCl_2_	0.0000285
Taurine	21.3
BSA	1 mg/mL
Collagenase type II (Worthington)	95 U/mL
Stop solution: Ca^2+^-free Tyrode plus	
CaCl_2_	0.0125
Newborn calf serum	0.06:1 mL
Storage solution ((Isenberg and Klockner, 1982), modified)	
KCl	25.0
KH_2_PO_4_	10.0
Glucose	20.0
L-aspartic acid potassium salt	10.0
L-glutamic acid potassium salt	100.0
MgSO_4_ x 7H_2_O	2.0
Taurine	20.0
EGTA	0.5
Creatine	5.0
HEPES	5.0
BSA (added on day of experiment)	1 mg/mL
​	stored at −8 °C, adjusted to pH 7.2 with KOH

**TABLE 2:** Stepwise re-exposition to Ca^2+^.

Ca^2+^-concentration (mmol/L)	Temperature condition
0.05	Cooled, tube on ice
0.1	
0.2	Spontaneous adaption to room temperature, no active heating
0.5	
1	

Stepwise exposition to Ca^2+^, 5 min acclimatisation period between steps.

### 2.5 Changes from standard (STD) to modified aCM isolation protocol (MOD)

The standard protocol (STD) was modified to include an additional cotton yarn ligature of the ventricle (MOD) as shown in Figure 1. The ligature was prepared as a loose double knot before animal sacrifice and kept submerged in the preparation solution. Immediately after fixation of the aorta on the cannula, it was positioned around the middle of the ventricle and then firmly tightened. The rest of the protocol remained unchanged.

### 2.6 Quantification and qualitative visual classification of isolated aCM

aCM suspensions were analysed at Ca^2+^ concentrations of 0 and 1 mmol/L with 3 technical replicates per isolation. For each technical replicate, 20 µL of the aCM suspension were incubated with 1 µL trypan blue (0.4%, Thermo Fisher Scientific Inc., Waltham, MA, USA) for 1 min to evaluate cell viability (Strober, 2015; Tennant, 1964). Then, 10 µL of the sample were transferred onto a slide and placed on the stage of an automated microscope (Nikon Ti-E, Tokyo, Japan). Several images covering the entire drop were taken at 20× magnification and merged. aCM were classified depending on the presence of trypan blue uptake and intact aCM morphology and counted. Intact aCM morphology was defined by typical cellular shape and absence of hypercontraction, rounding or “blebbing” (the formation of bubble-like membrane-protrusions) (Ackers-Johnson and Foo, 2019). The procedure is outlined with example pictures in Figure 1.

Additionally, images of exemplary aCM were acquired at 40× magnification.

### 2.7 Testing of aCM function and response to field stimulation

aCM isolated using the modified protocol were loaded with the Ca^2+^-sensitive dye Indo-1 AM and analysed using an IonOptix “calcium and contractility system” as described before (Schulte et al., 2016; Liffers et al., 2025). 50 μL of cell suspension were transferred to a custom organ bath and allowed to sediment before continuous superfusion with Tyrode’s solution containing 1 mmol/L Ca^2+^. The entire bath content was systematically screened for morphologically intact, Indo-1-positive aCM during field stimulation (0.5 Hz, 20 V, 20 ms). Ca^2+^ transients were recorded under baseline conditions and during stimulation with 1 μmol/L isoproterenol. Screening continued until at least 10 stable aCM with regular stimulation-induced Ca^2+^ transients were recorded under both conditions. The proportion of aCM that responded with stable stimulation-induced Ca^2+^ transients without spontaneous activity was determined.

### 2.8 Data presentation and statistical analysis

**FIGURE 4:**
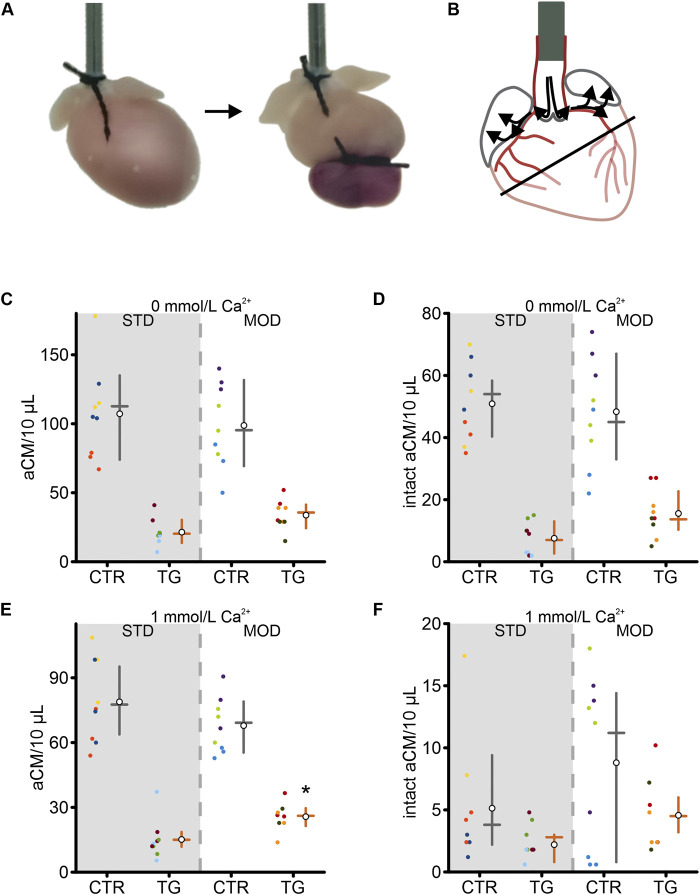
Ventricular ligature increases yield of Ca^2+^-tolerant aCM from remodelled atria **(A)** heart without (STD) and with ventricular ligature (MOD) visualising the modification of the established protocol. **(B)** Schematic hypothesis of changed myocardial perfusion by the ligature. **(C)** Total aCM/10 µL and **(D)** intact aCM/10 µL in absence of Ca^2+^(0 mmol/L): unchanged by MOD protocol in CTR hearts vs. STD, while there were tendencies to higher aCM yields with MOD protocol from TG atria. **(E)** Total aCM/10 μL at 1 mmol/L Ca^2+^: significantly higher total aCM extraction from TG atria with MOD vs. STD (p < 0.05). **(F)** Intact aCM/10 μL at 1 mmol/L Ca^2+^: tendency to higher counts of intact aCM both from CTR and TG atria with MOD vs. STD (n.s.). (n = 3 vs. 3, 3 technical replicates per isolation (raw data points, colour-coded per isolation), data for STD are identical to those shown in Figures 3C,D statistics on mean per isolation, t-test *p < 0.05 vs. STD). aCM: atrial cardiomyocytes, CTR: control group, TG: transgenic group (CREM-IbΔC-X mice), STD: standard isolation protocol, MOD: modified isolation protocol.

Numerical data are presented as mean ± standard deviation. In graphs, data are presented as boxplots (box: 25^th^-75^th^ percentile, whiskers: 10^th^-90^th^ percentile, horizontal line: median, filled circle: mean) or linerange (minimum to maximum, horizontal line: median, filled circle: mean) for samples n ≤ 3. Technical replicates were aggregated to means before statistical analysis and plot representation. Raw data are shown as overlaid points where applicable. To compare two independent groups, the unpaired two-tailed Student’s t-test was used. P values < 0.05 were considered statistically significant. Given the small number of independent isolations per group, statistical testing in Figures 3, 4 should be regarded as exploratory and as a model-based comparison of mean values. Data analysis and graphing were performed in RStudio/R statistical software (R Core Team, 2023; Posit Team, 2024).

## 3 Results

### 3.1 Extensive atrial remodelling in mice with heart directed CREM-IbΔC-X-overexpression

As demonstrated in earlier studies, TG mice displayed marked atrial remodelling by 12 weeks of age (Müller et al., 2005; Bukowska et al., 2018; Li et al., 2014; Stümpel et al., 2018; Kirchhof et al., 2013; Ni et al., 2022; Zhao et al., 2024). In explanted TG hearts, severe atrial dilatation was observed vs. CTR (Figure 2B: +217% vs. CTR, p = 0.00002, Supplementary Table S1). There were no substantial discrepancies in parameters such as body weight, tibia length, or ventricle weight between groups (Supplementary Table S2). The relative atrial weight was higher in TG compared to CTR (Figure 2D: +189% vs. CTR, p = 0.0000167). The histopathological examination illustrated enhanced atrial fibrosis, as previously described by our group (Figure 2F: +578% vs. CTR, p = 0.00327) (Kirchhof et al., 2013; Ni et al., 2022; Zhao et al., 2024).

In summary, TG atria exhibit substantial remodelling by 12 weeks of age, going along with an increase in connective tissue, which will likely compromise the process of aCM isolation.

### 3.2 Reduced aCM yield from remodelled, fibrotic atria

The standard protocol (STD) was suitable for isolating aCM from CTR hearts to assess the function of individual aCM in further experiments. The total aCM extract from CTR atria was 107 ± 31 aCM/10 μL, 49% ± 8% of which were morphologically intact. As expected, exposure to Ca^2+^ led to a reduction in the number of intact aCM. In the CTR group, the number of intact aCM was reduced by 90% to only 5 ± 4 intact aCM/10 µL. However, from TG hearts, the total aCM yield was 80% lower as compared to CTR (TG: 21 ± 8 aCM/10 μL, p = 0.0331, Figure 3C). 37% ± 23% of the acquired aCM showed intact morphology (8 ± 5 aCM/10 μL, −85% vs. CTR, p = 0.0053, Figure 3D). Here, the addition of 1 mmol/L Ca^2+^ to the aCM suspension reduced the number of intact aCM to 2 ± 1 aCM/10 µL (Figure 3D).

In summary, it is possible to isolate aCM using the STD protocol but the yield of intact aCM necessary for functional assessment was dramatically reduced when isolating aCM from remodelled TG atria.

### 3.3 Increased Ca^2+^-tolerant aCM extraction with ventricular ligature

The modified protocol (MOD) did not significantly affect the aCM counts from CTR atria (Figure 4, Supplementary Table S4).

In isolations from TG atria, there was a consistent tendency of higher aCM yields, both total and morphologically intact, with MOD compared to STD already at 0 mmol/L Ca^2+^ (Figures 4C,D). However, the total aCM yield from TG atria was significantly increased by 70% in MOD compared to STD at 1 mmol/L Ca^2+^ (Figure 4E, MOD vs. STD: 26 ± 4 vs. 15 ± 3, p = 0.0281). The number of intact aCM from TG atria was doubled in MOD vs. STD at both 0 and 1 mmol/L Ca^2+^ (Figures 4D,F, MOD vs. STD at 0 mmol/L: 16 ± 6 vs. 8 ± 5, p = 0.17; MOD vs. STD at 1 mmol/L: 5 ± 1 vs. 2 ± 1, p = 0.0931; see Supplementary Table S4 for details).

### 3.4 No additional information on cell viability revealed by trypan blue staining

We performed trypan blue-staining to assess cell viability. The count of aCM with intact morphology but intracellular uptake of trypan blue did not significantly differ between STD and MOD (Supplementary Table S1). Thus, this did not reveal relevant additional information as previously outlined by other groups (Nag et al., 1977; Dani et al., 1977). Furthermore, for the interpretation of the presented results the detrimental effects of trypan blue on cell viability should be kept in mind (Tsaousis et al., 2013). We therefore do not suggest the use of viability staining in routine isolation of CM for scientific purposes. A widespread practice of cell selection in single CM assessment is solely based on morphology and–when applicable-contractility (Cheung et al., 1984).

### 3.5 aCM showed regular Ca^2+^ transients and reliably responded to field stimulation

**FIGURE 5:**
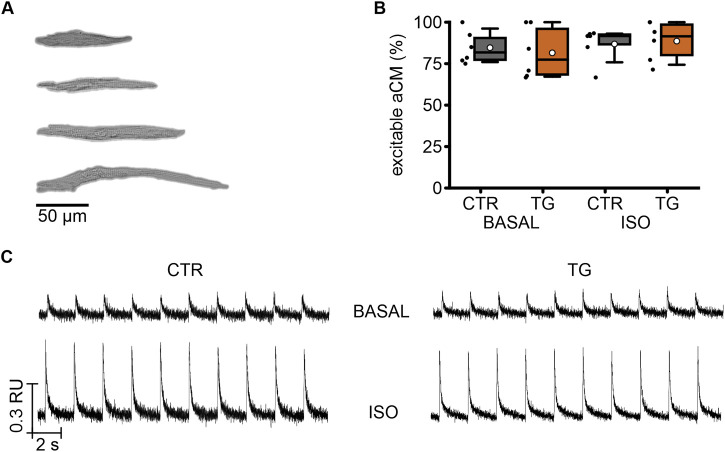
aCM respond well to electrical field stimulation **(A)** representative images of morphologically intact aCM isolated with the modified protocol. **(B)** Proportion of morphologically intact aCM analysed under the indicated conditions that responded reliably with field stimulation-induced Ca^2+^ transients without spontaneous activity. **(C)** Representative traces of Ca^2+^ transients, recorded under basal conditions (top) and during stimulation with 1 μmol/L isoproterenol (bottom) in CTR (left) and TG (right) aCM isolated with the modified protocol. (n = 6 isolations per genotype, 443 aCM in total, 12–34 aCM per data point shown in **(B)**). aCM: atrial cardiomyocytes, BASAL: basal conditions, CTR: control group, ISO: isoproterenol stimulation, RU: relative units, TG: transgenic group (CREM-IbΔC-X mice).

To assess the suitability of aCM isolated with the modified protocol for functional measurements we recorded Ca^2+^ transients in Indo-1 AM-loaded aCM during continuous field stimulation under basal conditions and during acute β-adrenergic stimulation with isoproterenol. Representative high-resolution images of aCM classified as morphologically intact demonstrate the typical cellular shape and cross-striation (Figure 5A). On average, 85% of aCM that were morphologically intact and exhibited regular dye loading responded with field stimulation-induced Ca^2+^ transients without visible spontaneous activity (Figure 5B; Supplementary Table S5). Representative Ca^2+^ transients are shown in Figure 5C both under basal and isoproterenol stimulated conditions.

## 4 Discussion

### 4.1 Isolation of murine CM is feasible with several available protocols

CM isolation is a critical method for the assessment of single cells in cardiovascular research. In general, perfusion-based approaches can be distinguished from chunk- or slice-based approaches. In the latter, tissue pieces are submerged consecutively in digestive solutions and a variety of protocols has been reported (Vahouny et al., 1970; Jansen and Rose, 2019; Fiegle et al., 2020; Greiner et al., 2022; Guo et al., 2018). An important advantage of chunk- or slice based protocols is their capability to isolate CM from discrete myocardial regions such as from the sinoatrial node (Sharpe et al., 2016; Aziz et al., 2020). Particularly for murine aCM, applicability of chunk- or slice-based isolation protocols might be limited by the lower quantity of atrial tissue in comparison to larger animals (ca. 4–5 g/atrium (Supplementary Table S2) vs. 20–30 g left atrium weight in rabbits (Guo et al., 2010). Furthermore, a lower CM yield from a non-perfusion-based compared to a perfusion-based protocol has been reported (Fiegle et al., 2020). This finding suggests that perfusion-based protocols might be superior.

As to our knowledge, the first adaptions of the retrograde Langendorff-perfusion (Langendorff, 1895) for the isolation of CM from mammalian hearts were described in the 1970s (Berry et al., 1970; Gould and Powell, 1972). Since then, a variety of modifications have been proposed to optimise this technique for isolation of CM, mostly focussed on vCM, from mice (Zhou et al., 2000; Fiset et al., 1997; Benndorf et al., 1985; Shioya, 2007; Tomsits et al., 2020; Louch et al., 2011). Due to the significant technical demands for Langendorff-perfusion - particularly in small model organisms - alternative perfusion methods have been proposed, for example, by intraventricular injection (Ackers-Johnson and Foo, 2019; Ackers-Johnson et al., 2016; Omatsu-Kanbe et al., 2018; Omatsu-Kanbe et al., 2021) or cannulation of the carotid artery (Jian et al., 2016), both in combination with a clamped aorta. However, establishment of the Langendorff-perfusion in the methodological portfolio comes with the advantage of a possible extension to a “working heart” experiment and its variety of experimental possibilities (Yang et al., 2024; Neely et al., 1967). The available literature predominantly focuses on vCM isolation, but there are separate protocols for aCM (Garber et al., 2022; Blackwood et al., 2020; Köhncke et al., 2013; Tomsits et al., 2020). Many groups report aCM isolation with vCM-established protocols (Omatsu-Kanbe et al., 2021; Omatsu-Kanbe et al., 2018) or even propagate simultaneous isolation (Wu et al., 2021; Blackwood et al., 2020). In our experience, simultaneous isolation is impracticable due to significant discrepancies in optimal digestion times for atria (30–32 min) and ventricles (6 min (Schulte et al., 2016)).

### 4.2 Atrial fibrosis is a hurdle to successful aCM isolation

In this study, we report significantly reduced total aCM yield and isolation quality in isolations from remodelled, fibrotic atria. Such atrial remodelling is also reported in other mouse models of atrial fibrillation (Hong et al., 2002; Xiao et al., 2004; Saba et al., 2005; Adam et al., 2007; Ikeda et al., 2009). In a study investigating the effects of aortic cannulation depth on simultaneous aCM and vCM isolation, Wu et al. (Wu et al., 2021) mentioned the limitations proposed by cardiac fibrosis to CM extraction. Bode et al. (Bode et al., 2018) also reported difficulties encountered in the presence of atrial fibrosis, albeit in a rat model of metabolic syndrome. Hence, we conclude that atrial remodelling complicates the isolation of aCM from rodent hearts, presumably depending on the extent of remodelling.

### 4.3 Altered coronary perfusion by ventricular ligature without prolonged heart preparation

For isolation quality, duration of preparation and (mechanical) stress on heart tissue are important factors (Jansen and Rose, 2019) and are required to be stable for reliable experimental series. In this study, the preparation time (from sacrifice to start of perfusion) was 5 min, which was comparable to reports for chunk-based isolation in murine atria (Jansen and Rose, 2019). Importantly, the additional ventricular ligature can be completely prepared before animal sacrifice and thus did not alter the duration of preparation (Supplementary Table S3).

Murine atrial coronary vessel branches are reported to be sparse and of small diameter (Kłosińska et al., 2020; Weyers et al., 2012; Fernández et al., 2007). Altered coronary perfusion in favour of the atria, for example, by compression of large ventricular coronary vessels, might result in higher exposure of atrial tissue to digestive solutions and explain larger aCM yields.

### 4.4 Higher counts of Ca^2+^-tolerant intact aCM in isolations with additional ventricular ligature

As mentioned before, due to the “calcium paradox” the number of Ca^2+^-tolerant CM is an important measure for the quality of a CM isolation (Powell, 1983; Zimmerman and Hülsmann, 1966). To avoid cell damage due to re-exposure to physiological Ca^2+^-concentrations after isolation, stepwise addition of Ca^2+^ is common practice (Zhou et al., 2000; Fiegle et al., 2020; Tomsits et al., 2020; Blackwood et al., 2020; Wu et al., 2021). It is widely accepted that aCM exhibit distinct Ca^2+^-homeostasis, and thus, it has been postulated that they are more susceptible to Ca^2+^-induced damage in comparison to vCM (Brandenburg et al., 2016; Greiner et al., 2022). Therefore, we evaluated the Ca^2+^-tolerance of aCM isolated with the proposed modified protocol. The present study demonstrated a relevant advantage for the modified protocol in isolations from remodelled, fibrotic atria, as it resulted in doubled numbers of intact aCM at 1 mmol/L Ca^2+^ (Figures 4D,F).

### 4.5 Implications for single aCM experiments in models of atrial remodelling

As evident in the extensive number of existing protocols, there are minor variations in perfusion, Ca^2+^ re-exposure or cannulation regimes, all of which are tailored to meet the specific requirements of the scientific question, model organism, target cell-subtype, laboratory and staff circumstances. To the best of our knowledge, there is no established protocol that addresses the difficulties that come along with the isolation of aCM from severely remodelled atria.

In this study, we propose a simple modification to a well-established, Langendorff-perfusion-based isolation protocol (Langendorff, 1895; Bode et al., 2018; Lemoine et al., 2011; Voigt et al., 2015; Motayagheni, 2017; Shioya, 2007; Wu et al., 2021; Garber et al., 2022; Tomsits et al., 2020) for aCM from murine hearts: a ventricular ligature to optimise atrial perfusion. Using a well-described model of atrial remodelling with CREM-IbΔC-X overexpressing mice (Müller et al., 2005, 2005; Kirchhof et al., 2013; Schulte et al., 2016; Seidl et al., 2017; Stümpel et al., 2018; Bukowska et al., 2018), we demonstrated that the modified protocol is superior for extracting aCM from remodelled, fibrotic atria for functional single-cell assessment. Morphologically intact aCM are commonly considered eligible for single-cell functional experiments (Cheung et al., 1984). The extracted number of such intact aCM from TG atria was consistently doubled per 10 µL although not statistically significant (p = 0.09). Considering the expenses and time investment required for single-cell functional assessment, this method clearly increases the success and cost-efficiency of experimental plans.

We routinely use the isolated cardiomyocytes in our laboratory for functional measurements such as calcium imaging and patch-clamp studies. We have not attempted to culture the isolated cells. Therefore, we are unable to determine whether the cardiomyocytes isolated using our modified method are suitable for long-term culture.

Single-cell functional experiments are susceptible to many confounding factors, including factors affecting the individual isolation (e.g., donor animal, person performing the isolation) (Sikkel et al., 2017; Park and Lake, 2005; Coppini et al., 2013). Investigating a greater number of aCM per isolation is vital to identify isolation-dependent confounding factors. Robust statistical techniques like hierarchical testing can help to reduce the chance of misinterpretation in large datasets, since these techniques consider the impact of individual isolations on the overall results (Sikkel et al., 2017). Thus, optimising aCM yield increases the reliability of findings and can help to reduce the number of laboratory animals required for experiments.

### 4.6 Conclusion

The addition of a ventricular ligature to an established aCM isolation protocol based on Langendorff-perfusion resulted in enhanced aCM isolation yields from fibrotic, remodelled murine atria. Consequently, this may facilitate research projects including single aCM assessment in murine models of severe atrial remodelling.

## Data Availability

The raw data supporting the conclusions of this article will be made available by the authors on reasonable request, without undue reservation.
