# Supplementary material for: A modification of retrograde Langendorff‐perfusion to increase the yield of Ca2+-tolerant murine atrial cardiomyocytes isolated from remodelled, fibrotic atria
Source: Front Pharmacol. 2026 Jul 8;17:1836453. doi: 10.3389/fphar.2026.1836453 (PMC13389159; doi:10.3389/fphar.2026.1836453)
Supplement: Supplementary file 1 [file DataSheet1.pdf]

Supplementary Material

1 Abbreviations

aCM: atrial cardiomyocytes, bw: body weight, CTR: control group, TG: transgenic group (CREM-IbΔC-X mice), STD: standard isolation protocol, MOD: modified isolation protocol (additional ventricular ligature)

2 Supplementary Tables

Table S1: Descriptives and statistics for atrial 2D-measurements

Genotype	n	atrial area (mm²)	ventricular area (mm²)
CTR	5	7.8±1.4	33.5±2.9
TG	5	24.6±2.8	33.8±1.3
p(t-test)	-	**0.0000222**	0.832

Table S2: Descriptives and statistics for body and heart morphometrics

genotype	n	bw (g)	atria (mg)	atria/bw (mg/g)	ventricles (mg)	ventricles/bw (mg/g)	tibia length (mm)	atrial fibr-osis (%)
CTR	6	25.6±3.0	9.02±1.82	0.35±0.03	101.2±15.0	3.95±0.29	17.4±0.5	4.3±2.3
TG	6	26.0±4.3	26.35±5.96	1.01±0.12	96.9±15.5	3.76±0.51	17.4±0.7	29.5±12.0
p(t-test)	-	0.871	**0.000519**	**0.0000167**	0.638	0.462	0.839	**0.00327**

Table S3: Experimental details

genotype	treatment	preparation time (s)	% visual atrial thrombosis	% visual dilatation
CTR	MOD	317±15	0	0
CTR	STD	330±79	0	0
TG	MOD	347±40	66,7	100
TG	STD	325±22	33,3	100

Table S4: Descriptives for isolation results from CTR and TG with STD and MOD protocol

genotype	treatment	Ca^{2+} (mmol/L)	n	aCM n/10 µL	intact aCM n/10 µL	% aCM intact/total	intact, trypan blue-stained aCM n/10 µL
CTR	STD	0	3	107±31	51±9	49±8	8±4
CTR	STD	1	3	79±16	5±4	6±3	3±3
CTR	MOD	0	3	99±31	48±17	48±2	3±1
CTR	MOD	1	3	68±12	9±7	12±10	3±2
TG	STD	0	3	**21±8***	**8±5***	37±23	2±1
TG	STD	1	3	**15±3***	2±1	20±8	1±1
TG	MOD	0	3	34±9	16±6	44±9	7±3
TG	MOD	1	3	**26±4#**	5±1	17±2	2±1
*p<0.05 vs. CTR, #p<0.05 vs. STD, t-test, no correction for multiple tests							

Table S5: Descriptives for Ca^{2+}-transient-responsiveness under field stimulation of aCM isolated from CTR and TG with MOD protocol under basal conditions and during acute stimulation with 1 µmol/L isoproterenol.

genotype	isoproterenol (µmol/L)	responsive aCM (%)	n animals	n aCM
CTR	0	84.6	6	108 (13-28/isolation)
CTR	1	86.9	6	94 (12-21/isolation)
TG	0	81.5	6	143 (16-34/isolation)
TG	1	88.6	6	98 (13-22/isolation)
